# An interview with Nobel Laureate David Baltimore, PhD

**DOI:** 10.20411/pai.v6i2.476

**Published:** 2021-08-30

**Authors:** Michael M. Lederman, Neil S. Greenspan

**Affiliations:** 1 Case Western Reserve University, Cleveland, Ohio

**Figure:**
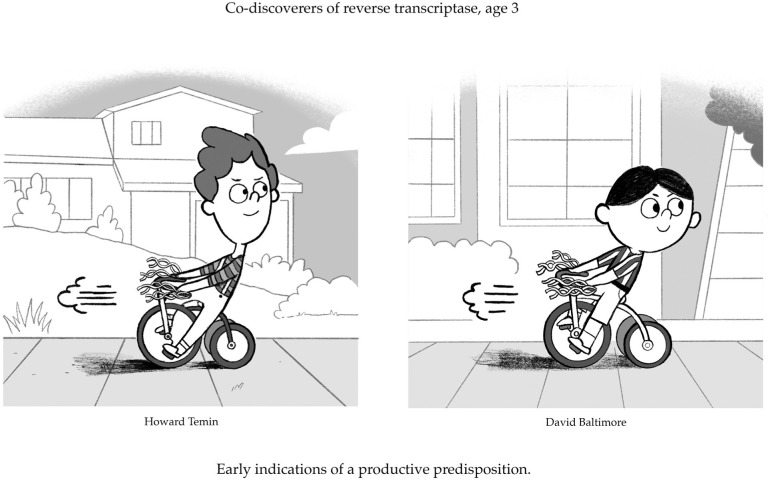


## MICHAEL M. LEDERMAN, MD

I am Michael Lederman. And with me is Neil Greenspan. We are editors of the journal *Pathogens and Immunity*, and we're here to have a conversation with Dr. David Baltimore. (Supplementary Video)

Dr. Baltimore is President Emeritus and Distinguished Professor of Biology at the California Institute of Technology. Dr. Baltimore received his undergraduate degree at Swarthmore and rapidly completed his PhD at The Rockefeller University.

After postdoctoral work in New York at The Rockefeller University and Albert Einstein, and a faculty position at the Salk Institute, he returned to MIT where he spent nearly 30 years and was founding director of the Whitehead Institute.

His contributions to biomedical science and science policy have been legion. His work has had major influence on the fields of molecular biology, virology, cancer, and immunology. He has trained numerous highly successful scientists who are leaders in their fields, and he helped to organize the Asilomar Conference on Recombinant DNA that established the guidelines for how to deal with recombining DNA.

He co-chaired the 1986 National Academy of Sciences Committee on a National AIDS Strategy and, in 1996, led the NIH (AIDS) vaccine research committee.

Among other notable works, he discovered the critical nuclear transcription factor NF Kappa B and the Rag1 and Rag2 proteins that rearrange adaptive immune cell receptor genes, and he and Howard Temin independently discovered reverse transcriptase revising the canon of cellular information transfer, for which they each received the Nobel Prize.

He is an accomplished researcher, educator, administrator, and public advocate for science and engineering and is considered one of the world's most influential biologists. Welcome Dr. Baltimore.

## DAVID BALTIMORE, PHD

Hello.

## ML

I'd like to start by asking you what early influences steered you to science in general and to virology in particular?

## DB

Well, I was steered to science because it came easily to me. And when I was in high school, mathematics and science classes were satisfying to me and were things I just naturally understood. And, so my mother, who was a scientist, recognized this and learned about a summer program at the Jackson Lab in Bar Harbor, Maine, and suggested I apply to it, which I did. And, so, between my junior and senior years of high school, I went to the Jackson Lab for the summer.

And that's where I discovered that the frontiers of science were not so distant; that I could actually make a discovery that nobody else in the world knew about. It was not a particularly important discovery, but it gave me the sense that doing experimental biology was fun, was rewarding. And I really set out to make the rest of my career as an experimental biologist.

## ML

Knowing something, learning, something that nobody else in the world knows is awfully seductive, isn't it?

## DB

It sure is.

## NEIL GREENSPAN, MD, PHD

Are you aware of any major differences in your thought process, your motivation to look for reverse transcriptase—or if that even was in fact what you were doing—or in the actual experimental approaches that you used - versus those of Howard Temin?

## DB

Not really. First of all, Howard had been arguing on the basis of his experimental work from the time when he was at Caltech as a graduate student that there was potential for their being an information transfer from RNA to DNA in the life cycle of RNA tumor viruses. But he was never able to find an experiment that convinced anybody else that was true when it was such a change in our thinking. It required one of those transparent experiments that convinces people easily.

I, on the other hand, was interested in the life cycle of RNA viruses as a molecular biologist and had studied polio virus and its relatives and some other viruses, and I recognized that the RNA tumor viruses either were very special and different than anything else I've worked on, or maybe were similar, but nobody had seen it.

And so I decided to have a look at them. And, actually, the first problem I had was getting a sample of RNA tumor virus to study, because I wanted to study it enzymologically. An old friend of mine, George Todaro, suggested that I might get some virus from a program at NIH that stored viruses for the day when somebody needed to work on them. And I called up the people who ran that program and said, I want to try this and they said fabulous and we'll send it to you, and they did, and I set up the experiment. And I had been doing enzymology on viruses and cells for my whole career at that point—10 years—following the in the footsteps really of Arthur Kornberg, although I didn't work with him, and I didn't know him. But he had established a paradigm of how you do an experiment of that sort, and so I just applied the methods that I had worked on so many other viruses with. And there was an enzyme in the virus particles that copied RNA to DNA.

## NG

So, I had heard that Max Delbrük was, I think, on Temin's thesis committee and expressed considerable skepticism about his claims. Do you know anything more about that story? I'm just curious; I guess he didn't do the same experiment you did—at least at that point in his career.

## DB

No. Actually, Howard had a postdoc when he was at the University of Wisconsin who did experiments very similar to the ones I did. And those were the papers that we published back-to-back in *Nature* [Baltimore; Temin, Mizutani]. But, when Howard was a student at Caltech, the idea that RNA might be copied to DNA was almost literally heretical, and Max did not believe it. It took our doing the enzymology 10 years later to convince Max and to convince the world that that was possible. Max had a tremendous admiration, I think, for Howard, and Howard had done really brilliant work for his thesis at Caltech.

## NG

Once you published those back-to-back papers, my impression is that the acceptance of this revolutionary concept was pretty quick.

## DB

It was basically instantaneous, and there were two reasons for that, I think.

One was that we had done these independent experiments, and so to doubt them, you'd have to doubt both his work and my work.

And the second thing is that we gave talks about the work and described it, and people went back to their labs and tested out the ideas and showed very quickly they could reproduce the experiments.

Famously, Sol Spiegelman, who had been working on the problem of the nature of these viruses for some years but had missed the idea that they might have a DNA intermediate; I think, on Friday Howard and I spoke at Cold Spring Harbor. Sol was there, went back to his lab, told the people, they worked through the weekend, Monday he came and said “We've reproduced it all.”

## NG

Wow. That's pretty quick.

## DB

Hey, it's easy to do once you have the idea of doing the right experiment.

## NG

I just think it's interesting in terms of the history of science, because some people have claimed that every revolution, you know doesn't catch on, and people have to die out, and that's not true here at all.

## DB

No, not in this case.

## ML

So, this enzyme, this reverse transcriptase, is central to the survival and life cycle of retroviruses and of course you've been actively engaged in much of the work and discussion and scientific directions in the HIV epidemic. What do you think will be an effective immunological approach to protection against acquiring HIV? Do you think we will be able to do this?

## DB

I am not sure whether we'll be able to make a vaccine. It is so elusive, so hard to pin down genetically that it's just not clear whether there can be a vaccine. If there is a vaccine, of course, that would be fabulous. I think we're more likely to live with HIV than to actually get rid of it.

## ML

So, we now have a whole array of functional neutralizing antibodies. What do you think of the future of vectored immunoprophylaxis for prevention of HIV?

## DB

I think that that is a methodology that might work. We've shown in experimental work with animals that we can design vectors that can be put into the muscle of an animal and will then pour out predefined monoclonal antibody and protect the animal against HIV or other viruses.

We have work in humans that has been reported at meetings which shows that we can use these vectors in humans, again by injection into muscle, to program antibody production.

It's independent of the immune system, being done much, as it turns out, like the COVID vaccine by getting the appropriate messenger RNA into muscle cells and they then pour out protein.

So, I think that could be harnessed as a mechanism of protection against HIV, and we're continuing to try to see if we can develop a company around that.

## ML

So, optimistic about vectored immunoprophylaxis for prevention, but uncertain about active immunization as a means to develop broad protection against HIV.

## DB

That's right.

## NG

Are you exploring cocktails, in other words, multiple different antibodies being produced simultaneously?

## DB

I assume that, in the long run, if we can get the methodology to work that the best way to use it would be making multiple antibodies.

## NG

And what sort of time frame, are you aiming at in terms of the length of time after injection that administration will continue to yield meaningful concentrations of antibody in the circulation?

## DB

What we know is that, in mice, we can do it for the lifetime of a mouse. So, a single injection to a 2-month old mouse will then protect the animal for a couple of years.

We also know that these kinds of vectors can produce proteins for as long as 10 plus years in humans—using viral vectors particularly.

## ML

In recent years there's been great emphasis on strategies to eradicate or provide functional cure. Are you optimistic that a scalable approach to cure or eradication is feasible?

## DB

I am not at all certain that there is a feasible way to completely eliminate HIV. It has a way of hiding inside cells in a latent form for years, and I haven't seen a methodology that will really clear it out.

There are a lot of people working on that. And it's a good idea to be hopeful, because that keeps people going, and maybe there is a particular way that you can rid the body of it, but I haven't seen evidence of that yet.

## ML

You've trained so many successful scientists; so many of your offspring have become leaders in the scientific enterprise. What do you think are the critical elements to being an effective scientific mentor?

## DB

I think first of all, that you have to believe in your students and give them as much freedom as possible to develop their own way of doing science, their own skills, their own interests, their own programs.

People don't usually give, particularly, students that freedom, even postdocs, and I think that hampers the development of young people. Because they have to do somebody else's experiment, not their own. And you learn from your own experiment, learn what does work, what doesn't work, you learn what interests you and what doesn't interest.

You have to be effectively a self-educator, and I tried very hard to provide the atmosphere, framework, opportunity for people to find themselves.

And I've seen it happen over and over again. It's not everybody who can take that kind of freedom and use it effectively. But the people who can, turn out to be the leaders of the next generation.

## NG

I recall that when you were directing Whitehead, you had developed a program where particularly promising PhD students could go straight into a sort of protected environment at Whitehead where they would run their own operation. Is that correct?

## DB

That is correct. We call them Fellows of the Whitehead Institute.

## NG

And, in retrospect, was that successful?

## DB

It's been enormously successful. And, you're quite right to ask about that, because I started that program because of my experience that people could handle independence much earlier than we give them the opportunity, and that program goes on today.

## NG

I think that's quite interesting in light of I don't remember the latest figures, but the last time I looked, the average age for first NIH award was like 43.

## DB

That's right. It's preposterous.

## ML

Should all MDs go to PhD school if they're serious about a research career?

## DB

They don't have to. There are many MDs who never got a PhD, but just went into research. My very good friend Irv Weissman being one of those. Arthur Kornberg was another one.

## ML

Is it practical nowadays?

## DB

The reward system and the hierarchy of professorships makes it really difficult. Because we look at credentials, and people who have a PhD just have that much more credentialing, so it's really difficult, and I don't see young people with only an MD these days getting into research. It's just harder for them to get into labs to get the background. A lot of the most successful research MDs went through NIH, went through the (Associates Training Programs), and those don't exist anymore.

## ML

You were critically engaged in the early discussions about the hazards of working with recombinant DNA, and now there's a lot of discussion and reservation about gain-of-function research with regard to pathogens. How should we be approaching these issues scientifically and safely?

## DB

I think we have to be very honest with ourselves about what might hold danger.

We have to control our instinct, which is to just do anything we can to generate progress and understanding of life. And there are certain things we may have to hold back because they do create danger and that's what we're learning from the COVID pandemic - the danger in the natural world. And so, we have to be careful.

At the same time, we don't want to hold back progress and so there is a balancing; you have to be careful.

## NG

Do you have strong opinions about the rise of these Bioarchives, Medicalarchives of pre-prints, and whether that's good or bad for scientific publishing? These are not yet peer-reviewed manuscripts.

## DB

When I was a young scientist, we had them. There was a program. I guess it must have been NIH-sponsored, and people just naturally gave their pre-prints to this program. As it got larger and larger, it became very expensive and ultimately was stopped. But I thought it was a terrific program, because you got a look at science at the earliest possible time in its publication; when it's first submitted. And it just made progress faster, it oiled, other people's experimentation, and allowed them to take advantage of the most recent information.

I think it's wonderful.

It gets in the way of other problems. It often holds information, which turns out to be wrong. It holds the information in a form which may not be rigorous. And so, you have to be careful. But I think it's a good thing.

## ML

In an era where NIH funding is increasingly tight, increasingly difficult, it's increasingly difficult to distinguish among the strong applications that are reviewed by study section. And so, in this very difficult time, how do we balance the role of targeted funding in response to notices by the NIH versus investigator-initiated original research that is not responsive to an RFA, for example.

## DB

Well, you know, I'm a great believer in the creative force of young investigators.

I think funding has to be largely directed to people and ideas that are generated by the field of investigators. They don't necessarily have to be young.

## ML

They could be 43 years old.

## DB

They could be 43 years old. They could be 55. They could be even 80. But the people who run the programs at NIH don't necessarily have the best ideas. And so, their programs may be good and they may, particularly if they are designed around the transition of information from the basic lab to practical utilization, they may actually be very valuable. But you have to balance that off against keeping basic research going.

## ML

So if you didn't get drawn immediately to a career in science, what would you have done?

## DB

You know, I don't know what I would have done. There's nothing that I've ever felt I was particularly good at outside of experimental biology, and so there's nothing that has attracted me to try. I like writing. I really enjoy writing, but I don't feel that I have the creativity of a, particularly, a fiction writer.

## ML

Well, we're glad you stayed with nonfiction. I have one last question to ask you. My last question is: Do you still play the tuba?

## DB

No I don't.

## ML

Should we be happy that you don't or should we be sad?

## DB

I was never great. I played the tuba into my first year in college. And then in college, I was in the college orchestra, and they did a Stravinsky piece, and I could never come in on the offbeat. And I realized that there was no hope for me as a musician.

## ML

Well that's a great way to end this discussion. Dr. Baltimore, thank you so much for being with us. This was just fantastic.

## CONFLICTS OF INTEREST

The interview was conducted via Zoom by Michael M. Lederman and Neil S. Greenspan. Michael M. Lederman and Neil S. Greenspan are professors at Case Western Reserve University. Dr. Lederman is also the editor-in-chief of *Pathogens and Immunity*. Neil S. Greenspan is a Senior Editor for *Pathogens and Immunity*.

## SUPPLEMENTARY DATA

Supplementary materials are available at the *Pathogens and Immunity* website. Supplementary data may be provided by the authors to benefit the reader. Supplementary data are not copyedited and are the sole responsibility of the authors. Questions or comments related to supplementary materials should be addressed to the corresponding author.

Supplementary Video

